# Gender differences in behavioral inhibitory control under evoked acute stress: An event-related potential study

**DOI:** 10.3389/fpsyg.2023.1107935

**Published:** 2023-03-07

**Authors:** Siyu Di, Chao Ma, Xiaoguang Wu, Liang Lei

**Affiliations:** ^1^Normal College, Shihezi University, Shihezi, China; ^2^Center of Application of Psychological Research, Shihezi University, Shihezi, China

**Keywords:** acute stress, behavioral inhibition, gender, oddball, event-related potential

## Abstract

**Purpose:**

This study investigated gender differences in behavioral inhibitory control among college students under acute stress state by using event-related potential technique.

**Methods:**

Acute stress was evoked in 41 college students (22 males and 19 females) using the Trier Social Stress paradigm, and the neutral state was matched using out-of-speech reading, with subjects completing a two-choice Oddball task in each of the two states. In combination with the ERP technique, the area under the stress curve, reaction time, number of errors, and the difference waves between the two stimulus conditions in the frontal-central region N2 wave amplitude and the parietal-central region P3 wave amplitude were compared between the two groups of subjects in the stressful and neutral state.

**Results:**

The results revealed that the area under the stress curve was larger under the stress condition compared to the neutral condition, and the area under the stress curve was larger in females than in males. Behavioral results showed no statistically significant differences in reaction time and number of errors between the two genders in the acute stress condition. The ERP results showed that the wave amplitudes of N2 and P3 decreased significantly in both genders in the acute stress state. The decrease in N2 amplitude was greater in females during the transition from neutral to stressful condition, while the difference in P3 amplitude was not statistically significant in both genders.

**Conclusion:**

The findings suggest that evoked acute stress can promote behavioral inhibitory control in both genders and that females are more sensitive to acute stress state.

## 1. Introduction

Behavioral inhibitory control, also known as response inhibition, is one of the core components of executive functioning (Miyake and Friedman, 2012). Behavioral inhibitory control refers to people’s inhibition of their inappropriate external behaviors under specific environment conditions, such as resisting external temptations and suppressing impulsive behaviors (Puiu et al., 2020). From a cognitive perspective, behavioral inhibitory control includes early perceptual processing, conflict awareness, and late response inhibition (Yuan et al., 2008). With better behavioral inhibition control, individuals can monitor and suppress their current or upcoming inappropriate behaviors, effectively guiding them to adopt corresponding behavioral patterns in response to changes in the environment and ultimately make correct behavioral decisions (Goldstein and Tuescher, 2007). The lack of behavioral inhibitory control often leads to a series of problems. On the one hand, reduced behavioral inhibitory control may lead individuals to uncontrolled violent behavior, delinquency, and suicidal behavior. On the other hand, weaker behavioral inhibitory control is also detrimental to the development of physical health. Some studies have found that most obese patients cannot control their diet because of their low behavioral inhibition control, which eventually leads to obesity. At the same time, as further research has been conducted, researchers have found that some disorders are also associated with behavioral inhibition control, such as attention deficit hyperactivity disorder, depression, obsessive–compulsive disorder, and schizophrenia (Hatta et al., 2001; Kelly et al., 2020; Zhao et al., 2020). Therefore, the importance of behavioral inhibitory control for individual development cannot be overstated.

It has been shown that there may be gender differences in behavioral inhibitory control. As an important executive function, behavioral inhibitory control is critical to the success of both males and females in modern society and may have played a key role in the evolution of human social intelligence (Li et al., 2006). Sjoberg et al. used the Go/No-go paradigm to examine gender differences in behavioral inhibition and found that female exhibited better behavioral inhibition (Sjoberg and Cole, 2018). However, when Melynyte et al. (2017) used the same method for their study, they found that females were less correct and required more time for conflict monitoring and response inhibition, suggesting that females have worse behavioral inhibition. Some other researchers have argued that there are no gender differences in behavioral inhibitory control. For example, Herba et al. (2006) examined changes in behavior inhibitory control using the Go/No-go paradigm and found no gender differences (Yuan et al., 2010). Neuroimaging findings were similarly divergent, with Liu et al. (2012) finding more statistically significant activation of the left sub-parietal and striatal regions in behavioral inhibitory control processing in females, while males showed greater activation of the right sub-parietal and suprachiasmatic regions, as well as stronger anterior cingulate gyrus activation. In contrast, other studies have not found similar statistically significant lateralization features (Garavan et al., 2006; Liu et al., 2012). In summary, the available studies have not clarified whether there are gender differences in behavioral inhibitory control, presumably because they may be influenced by factors such as research paradigms and situational factors.

It has been found that behavioral inhibitory control is also influenced by acute stress. Stress is a series of physiological and psychological reactions of the organism to maintain homeostasis when its internal steady state is threatened (Zhenzhen et al., 2017). Acute stress is a part of everyone’s life, and there are many sources of acute stress in life. In daily life, individuals often face various threats and challenges, such as sudden exams or interviews, various public emergencies, and so on. In the face of acute stressors, the body’s internal homeostasis is rapidly unbalanced and can trigger a series of physiological stress responses. The results of the effects of acute stress on individual behavioral inhibition remain divergent. Some studies have shown that acute stress impairs individuals’ behavioral inhibition (Jiang and Rau, 2017; Roos et al., 2017). When Jiang et al. used the Trier Social Stress Test (TSST) to study behavioral inhibitory control in individuals, they found a statistically significant rise in reaction time and an increase in P3d volatility in the stress group. However, other studies have suggested that acute stress elevates the behavioral inhibition capacity of individuals (Farbiash, 2016; Qi et al., 2017; Dierolf et al., 2018). Dierolf used the Trier Social Stress Test (TSST) paradigm to evoke different age males in an acute stress state, followed by testing the subject’s behavioral inhibitory control using the Go/No-go task, and found shorter inhibition time and smaller N2d wave amplitudes in the stress state (Dierolf et al., 2018). In summary, the direction of effect of acute stress on behavioral inhibitory control in individuals has not been clarified by existing studies. Furthermore, currently, gender differences in behavioral inhibitory control under acute stress have not been directly explored.

In this study, we chose a modified two-choice Oddball paradigm to evoke behavioral inhibitory control in subjects (Yuan Jiajin et al., 2017) and combined it with the event-related potentiation technique, which is known for its high temporal resolution, to explore gender differences in behavioral inhibitory control under acute stress state. In the two-choice Oddball task, subjects are required to respond to two types of stimuli, one type is the standard stimulus with a high number of occurrences and corresponding responses. The other category is the deviant stimulus, which occurs less frequently and corresponds to fewer responses. Subjects were required to respond to both types of stimuli with keystrokes. The time difference between the responses to standard and deviant stimuli is used as a behavioral inhibition index, which effectively resolves the interference of motor contamination on the electrophysiological results existing in the Go/No-go task and the stop signal task (SST). ERP has a high temporal resolution and is often used to examine the time course of behavioral inhibitory control. Among the ERP components, the main focus is on two components, frontal-central N2 and central-parietal P3. N2 is a negative component that usually appears around 200 ms after stimulus presentation, and the maximum wave amplitude generally occurs in the prefrontal region. N2 emerges in the early stage of behavioral inhibitory control and mainly reflects conflict monitoring and conflict control. The change in N2 wave amplitude is related to conflict monitoring ability (Donkers and Boxtel, 2004; Dong et al., 2009). P3 is a positive component that usually appears around 300 ms after stimulus presentation, with the maximum wave amplitude generally appearing in the central parietal lobe. P3 emerges in the late stage of behavioral inhibitory control, mainly reflecting the inhibition process itself and related to the completion of the inhibition process. The change in P3 wave amplitude is related to the degree of cognitive effort invested (Donkers and Boxtel, 2004; Dong et al., 2009). We expected to see a moderating effect of gender on the amplitude of the N2 and P3 components, and this variation reflects the changing process of behavioral inhibitory control.

It has been shown that acute stress can affect behavioral inhibitory control and that gender differences may also have an impact on behavioral inhibitory control. However, the direction of the effect of acute stress on behavioral inhibitory control in gender-specific individuals is still unclear. Investigating the gender differences in behavioral inhibitory control under acute stress can help us understand the characteristics and discrepancies in behavioral inhibitory control between the two genders in the face of stress, which in turn can help us provide targeted strategies to enhance behavioral inhibitory control. In addition, it is especially helpful to help both genders of college students to have higher behavioral inhibitory control when facing the stress in current society, dealing with various problems in life calmly, resisting temptations better, and making correct behavioral decisions. Based on existing studies, this study hypothesized that acute stress would motivate individuals to respond positively, which in turn would enhance their behavioral inhibitory control, as evidenced by a decrease in the time of behavioral inhibition and a reduction in the number of errors under acute stress. Furthermore, we hypothesized that behavioral inhibitory control is more susceptible in females than in males under acute stress state.

## 2. Materials and methods

### 2.1. Participants

*A priori* analysis was completed using G*Power 3.1 software (effect size *f* = 0.3, *α* = 0.05, 1−*β* = 0.80, repeated measures, 2 between-group*2 within-group), and calculations showed that a total of 24 subjects (12 in each group) were required. The convenience sampling method was adopted to recruit 44 university student subjects, 22 females and 22 males, through a recruitment announcement on campus. Subject selection criteria: age 18–25, right-handed, without major physical illness, no history of neurological or psychiatric disorders, no previous participation in relevant trials, non-restricted dieters, no color weakness or color blindness, body mass index in the normal range (18.5–23.9), and normal visual acuity or more positive visual acuity. Subject exclusion criteria: scores (26.1 ± 3.8) higher than 48 (moderate anxiety or higher) on the Trait Anxiety Inventory (Dai, 2014) and scores (6.7 ± 3.1) higher than 14 (moderate depression or higher) on the Beck Depression Inventory (Jackson-Koku, 2016). After the experiment, one subject failed to record all data due to an instrument error, and the other two subjects were deleted due to excessive signal noise caused by physical activity during the trial, resulting in an insufficient number of valid trials. 41 participants were actually enrolled, including 22 males and 19 females, aged 18–25 years, with a mean age of (20 ± 2) years. The study was in accordance with the Declaration of Helsinki, was reviewed and approved by the local medical ethics committee, and the subjects voluntarily participated in the experiment and signed the informed consent form.

### 2.2. Materials

#### 2.2.1. Subjective measurement

The Short State Anxiety Inventory (Marteau and Bekker, 1992) measures an individual’s state anxiety. The scale includes a total of eight entries, including sad, disgusted, angry, distracted, nervous, upset, relaxed, and calm. The scale is scored on a seven-point scale from 1 (very nonconforming) to 7 (very conforming), with the last two items scored inversely, and a higher total score represents a higher level of state anxiety.

#### 2.2.2. Stress-evoking

The study used the TSST paradigm to evoke an acute stress state, which consisted of two parts: free speech and mental arithmetic (Kirschbaum et al., 1993). In the acute stress state, subjects were simulated to participate in a multi-competitive recruitment event. Subjects were given 2 min to organize their language and then completed a self-presentation of about 5 min. When subjects had less than 5 min for self-presentation, each of the three main testers asked subjects about the prepared questions. The entire presentation was recorded. After completing the free speech task, the subjects were asked to complete the mental calculation task of subtracting 17 from 2023 in succession, without giving feedback if the calculation was correct and reminding the subjects to stop and start again from 2023 if the calculation was incorrect. The two states were balanced between subjects.

#### 2.2.3. Task

**Figure 1:**
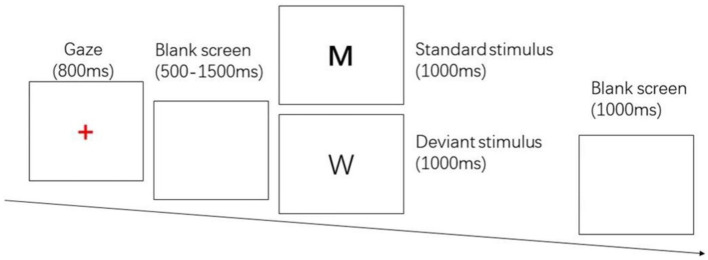
Two-choice Oddball task.

The two-choice Oddball task evoked behavioral inhibition, and the stimulus materials were the letter pictures “W” and “M.” “M” was the standard stimulus, press the “F” key; “W” was the deviant stimulus, press the “J” key. The whole test procedure was prepared by E-prime 2.0, and the stimuli were presented on a DELL 23-inch LCD monitor with a picture size of 356 pixel × 391 pixel, and the subject’s eyes were about 80 cm from the center of the screen. 280 trials were included in the test, including 200 standard stimuli and 80 deviant stimuli. First, a red “+” gaze point appears in the center of the screen for 800 ms, followed by a random blank screen for 500 ~ 1,500 ms, then a random standard stimulus/deviant stimulus with a presentation time of 1,000 ms, the subject needs to respond correctly in time, and after the keystroke ends, there will be a blank screen for 1,000 ms. The entire process used E-prime 2.0 to record response time and number of errors. See Figure 1.

### 2.3. Procedure

**Figure 2:**
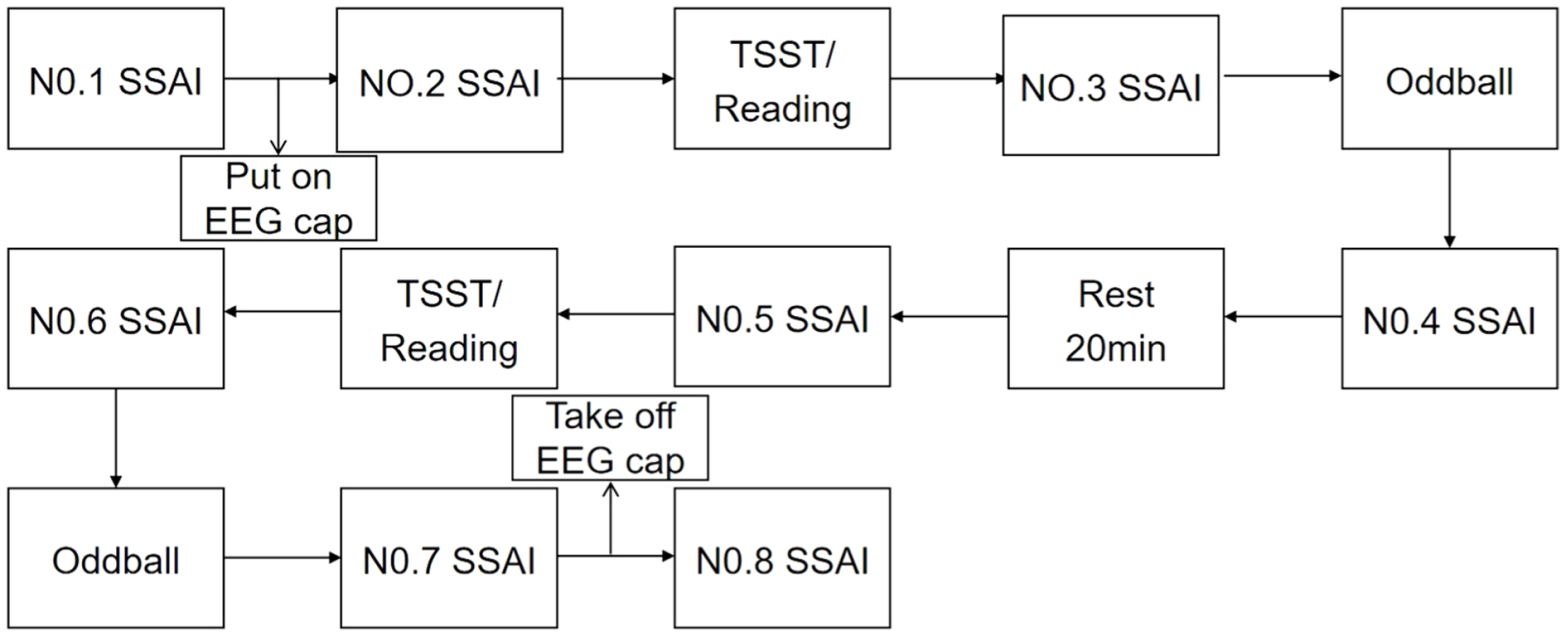
Experimental flow chart.

Participants were contacted 1 day in advance and told not to exercise and not to eat for 2 h prior to the test. Subjects were asked verbally prior to the test whether they had met the above requirements. Upon arrival at the laboratory, subjects first washed their hair and sat quietly for 20 min, then filled in their personal information and administered the 1st SSAI. Subsequently, after wearing the equipment and completing the practice trials, the 2nd SSAI was administered. Afterwards, the TSST paradigm/reading was performed for 15 min, and the 3rd test was administered. Following the stress/neutral state evocation, the Oddball trial task was completed, EEG data were recorded, and the 4th measurement was taken after the task was completed. After a 20 min rest, the 5th measurement was taken. Afterwards, TSST/reading was performed, and the 6th measurement was taken, the Oddball trial was completed, EEG data was recorded, and the 7th measurement was taken afterwards. The 8th measurement was administered after the completion of all test tasks and the end of hair washing. The 1st measurement was used as a baseline for mood, and the last measurement was used as a recovery of mood after completing the task. The results of the intermediate 6 Measurement were used to assess the status of the subject and also to calculate the area under the stress curve for both genders. See Figure 2.

### 2.4. Data recording and analysis

The EEG signal was collected using the Neuroscan EEG collection system. The EEG cap was a 64-conductor cap. The EEG data were collected using Curry7 software and the mean values of bilateral mastoids (M1, M2) were used as a reference. At the beginning of the experiment, the resistance between all electrodes and the scalp was less than 10kΩ. The EEG data was collected in DC mode at a sampling frequency of 1,000 Hz/conductor and was filtered online by a DC-100 Hz bandpass filter at the beginning of the experiment. After continuous data collection, the data was processed off-line using eeglab13.0. Waves below 0.05 Hz and above 30 Hz were removed by eeglab13.0. The sample rate was reduced to 500 Hz/conductor. The segmentation was performed at 200 ms before and 800 ms after the spike, with the spike occurrence as the zero point. After the segmentation, artifacts such as eye-electricity were removed using independent component analysis (Garber et al., 2011), and then extreme values with voltages greater than ±100 μV were removed. Finally, all remaining segments were superimposed and averaged to calculate the difference waves between the two stimulus conditions.

According to previous studies, behavioral inhibition is mainly associated with frontal areas (Gaertner et al., 2015). Therefore, N2 values were chosen as the mean of (F1, FZ, F2) three electrode sites and P3 values were chosen as the mean of (P1, PZ, P2,) three electrode sites. In order to separate out the inhibitory control components, the ERPs under the two stimuli were subtracted (deviant-standard) to obtain the difference waves between the two stimulus conditions. 225-275 ms was chosen as the time window for N2 according to previous studies (Rueda-delgado et al., 2021). The time window of P3 was chosen as 350-500 ms (Alatorre-Cruz et al., 2021).

The study used the SSAI scale score as an indicator of stress, and a repeated measures ANOVA of 2 (gender: males, females) × 2 (state: stress, neutral) was performed on reaction time and number of errors. The measures conformed to a normal distribution and were expressed as mean ± standard deviation, and the *p* values of all repeated measures ANOVAs were Greenhouse spherical corrected, and statistical analysis was performed using SPSS 26.0 software.

## 3. Results

### 3.1. Subjective measurements

The area under the stress curve was subjected to repeated measures ANOVA for males and females in the acute stress state and the neutral state. The results showed that the state main effect [*F*(1,39) = 68.96, *p* < 0.001, *ηp^2^* = 0.64] was statistically significant for scores on the SSAI scale, and the area under the stress curve was larger for both genders in the acute stress state than in the neutral state. The gender main effect [*F*(1,39) = 0.87, *p* > 0.05, *ηp^2^* = 0.02] was not statistically significant. The interaction between state and gender was statistically significant in the score [*F*(1,39) = 12.04, *p* < 0.05, *ηp^2^* = 0.24]. The area under the stress curve for females was greater than the area under the stress state curve for males. See Table 1.

**Table 1:** Comparison of the area under the subjective mood score curve in males and females in acute stress and neutral state [(*x* ± *s*)].

	Females	Males	*F*	*P*	*ηp^2^*
Neutral	20.66 ± 1.08	22.21 ± 1.16	0.97	0.332	0.02
Acute stress	30.59 ± 1.23	26.29 ± 1.33	5.66	0.022	0.13
*F*	74.78	10.89			
*P*	<0.001	0.002			
*ηp^2^*	0.66	0.22			

### 3.2. Behavior results

A 2 (state: stress, neutral) × 2 (gender: males, females) repeated measures ANOVA was conducted for reaction time and number of errors for the Oddball experiment, respectively.

Concerning response time, the state main effect [*F*(1, 39) = 4.45, *p* < 0.05, *ηp^2^* = 0.10] was statistically significant, with longer reaction time for both genders in the neutral state than in the stress state. The gender main effect [*F*(1, 39) = 4.41, *p* < 0.05, *ηp^2^* = 0.10] was statistically significant, with longer reaction time for females than for males in both the neutral and stress state. The interaction of state and gender [*F*(1, 39) = <0.01, *p* > 0.05, *ηp^2^* < 0.01] was not statistically significant. See Table 2.

**Table 2:** Comparison of response time between males and females in acute stress and neutral state [(*x* ± *s*)].

	Females	Males	*F*	*P*	*ηp^2^*
Neutral	124.24 ± 14.31	96.25 ± 13.30	2.05	0.160	0.05
Acute stress	105.83 ± 7.47	78.89 ± 6.94	6.97	0.012	0.15
*F*	2.20	2.25			
*P*	0.146	0.141			
*ηp^2^*	0.05	0.06			

Concerning the number of errors, the state main effect [*F*(1, 39) = 11.73, *p* < 0.05, *ηp^2^* = 0.23] was statistically significant, with both genders in the stress state having fewer number of errors than in the neutral state. The gender main effect [*F*(1, 39) = 4.44, *p* < 0.05, *ηp^2^* = 0.10] was statistically significant, with females making more errors than males in both the neutral and stress state. The interaction of state and gender [*F*(1, 39) = 1.93, *p* > 0.05, *ηp^2^* = 0.05] was not statistically significant. See Table 3.

**Table 3:** Comparison of number of errors in males and females in acute stress and neutral state [(*x* ± *s*)].

	Females	Males	*F*	*P*	*ηp^2^*
Neutral	6.14 ± 0.62	5.63 ± 0.66	0.31	0.581	<0.01
Acute stress	5.00 ± 0.50	2.95 ± 0.54	7.87	0.008	0.17
*F*	2.24	10.79			
*P*	0.143	0.002			
*ηp^2^*	0.05	0.22			

### 3.3. ERP results

The EEG data from the Oddball task were subjected to a 2 (state: stress, neutral) × 2 (gender: males, females) repeated measures ANOVA, and the statistics were corrected for *p*-values using the Greenhouse–Geisser correction. A statistical significance level of <0.05 was chosen for statistics, and *ηp^2^* was used for statistical effect values, and Bonferroni-adjusted correlations were chosen for *post hoc* comparisons.

#### 3.3.1. N2 (225–275 ms)

**Figure 3:**
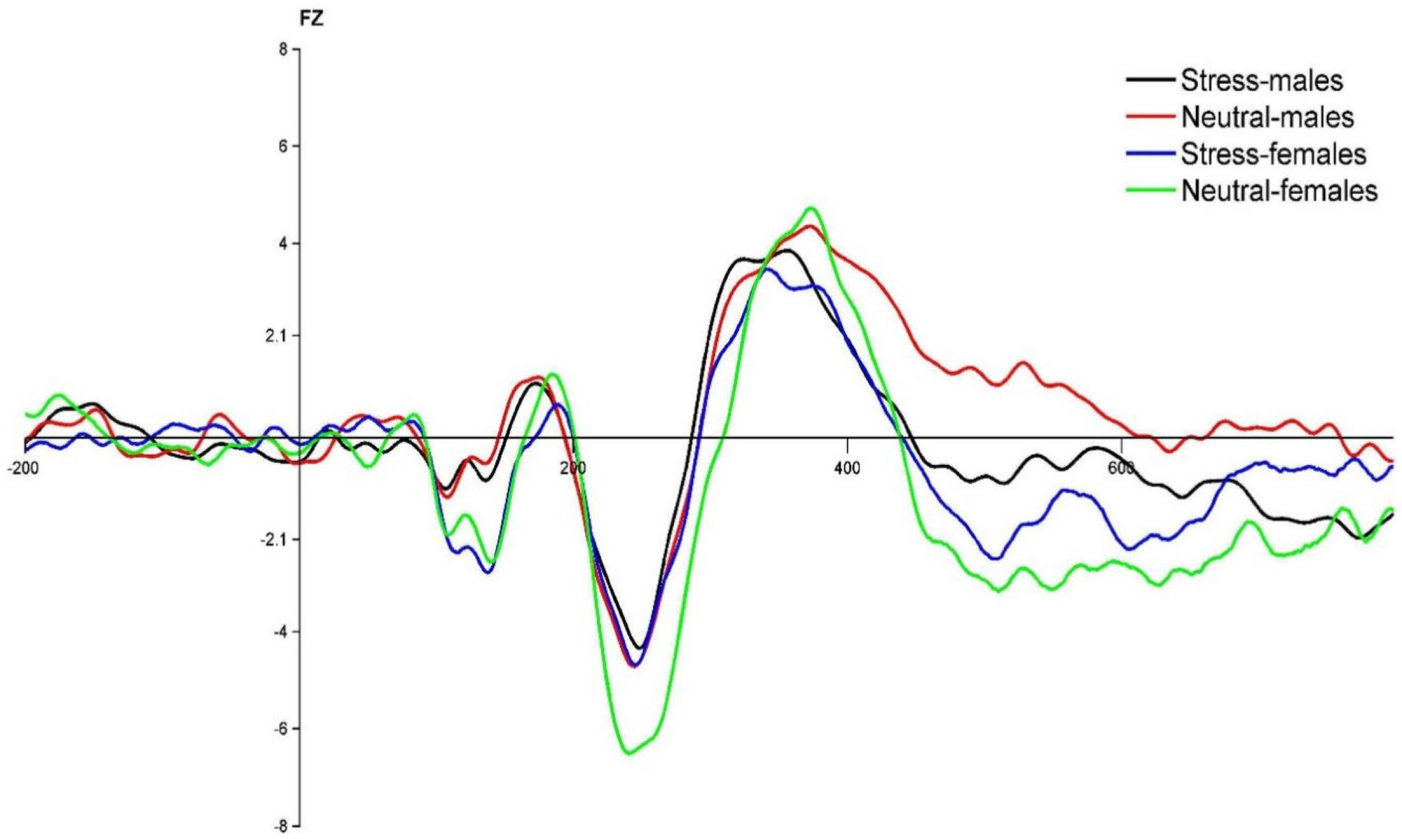
FZ point difference waveform (deviant-standard) in males and females in acute stress and neutral state.

The state main effect [*F*(1, 39) = 7.14, *p* < 0.05, *ηp^2^* = 0.16] was statistically significant. The N2 wave amplitude in both males and females was smaller in the stress state than in the neutral state. The state and gender interaction [*F*(1, 39) = 4.28, *p* < 0.05, *ηp^2^* = 0.10] was statistically significant. *Post hoc* tests comparing the two states revealed greater changes in the amplitude of the N2 wave in females compared to males. The gender main effect [*F*(1, 39) = 0.90, *p* = 0.40, *ηp^2^* = 0.02] was not statistically significant. See Table 4 and Figure 3.

**Table 4:** Comparison of difference wave (deviant-standard) N2 amplitudes in males and females in acute stress and neutral state [(*x* ± *s*) μν].

	Females	Males	*F*	*P*	*ηp^2^*
Neutral	−5.99 ± 1.21	−3.07 ± 1.13	1.69	0.201	0.04
Acute stress	−2.10 ± 1.02	−2.58 ± 0.94	0.41	0.528	0.01
*F*	5.26	0.78			
*P*	0.027	0382			
*ηp^2^*	0.12	0.02			

#### 3.3.2. P3 (350–500 ms)

**Figure 4:**
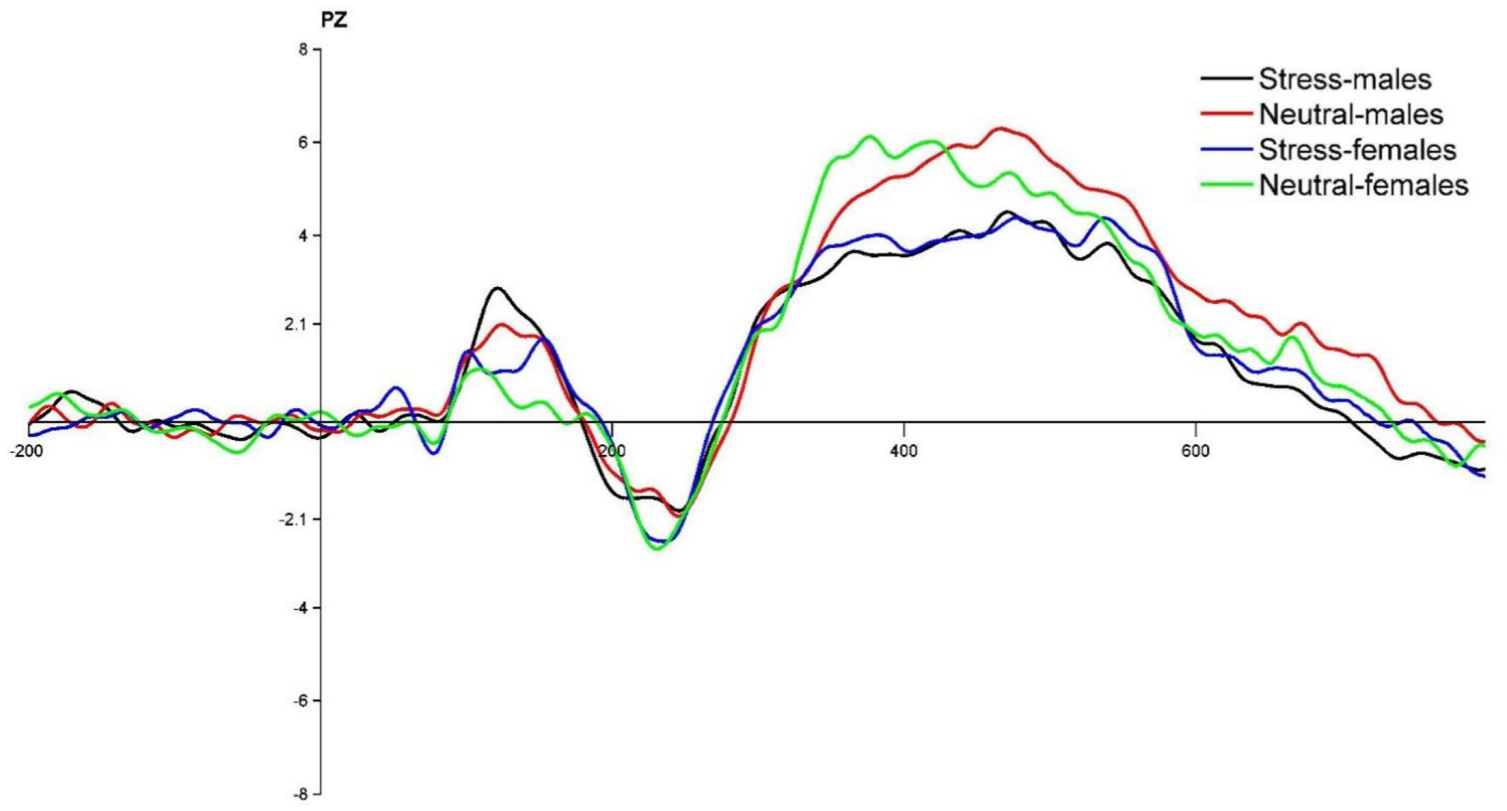
PZ point difference waveform (deviant-standard) in males and females in acute stress and neutral state.

**Figure 5:**
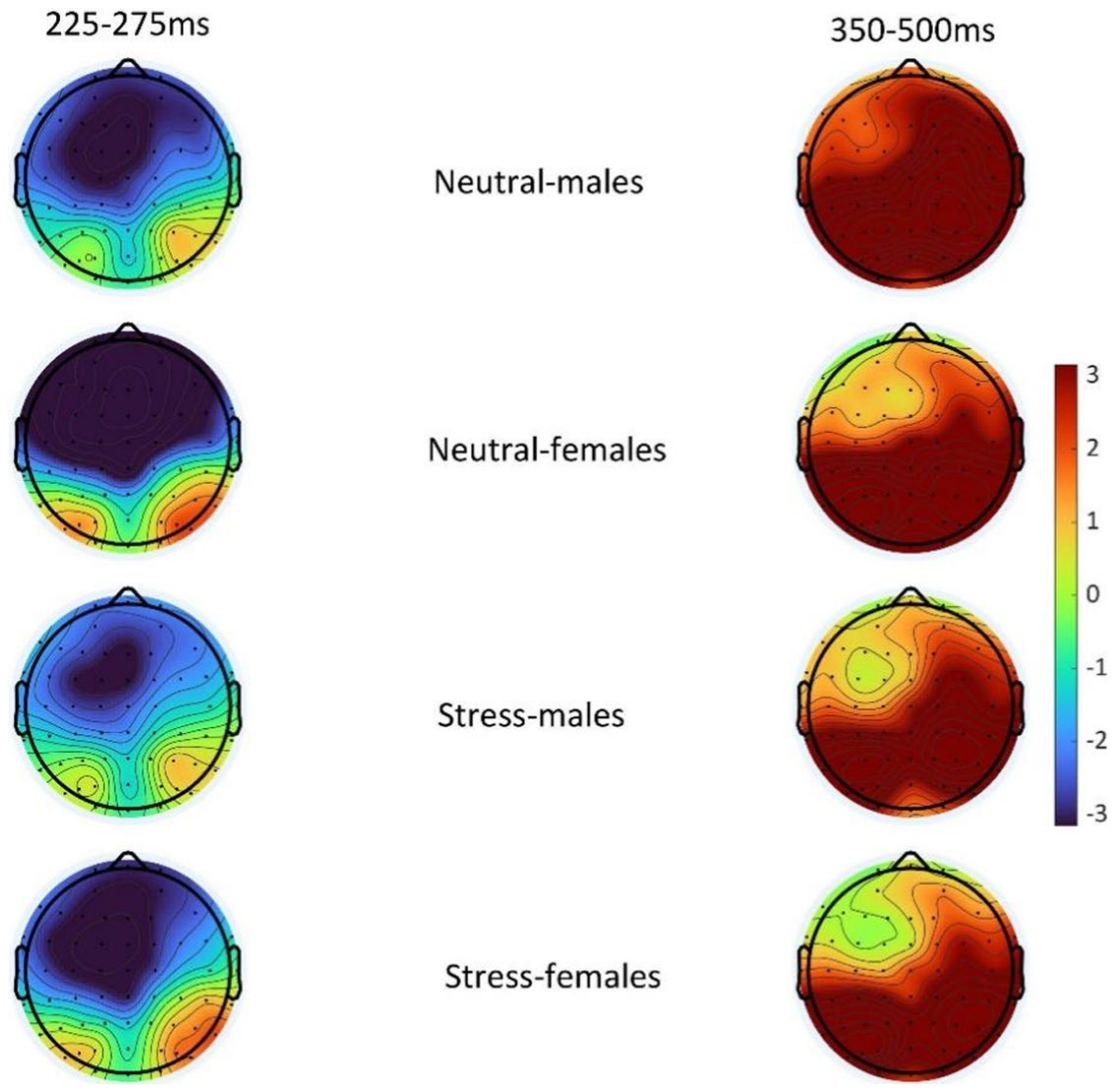
Topography of difference wave (deviant-standard) N2 (225–275 ms) and difference wave (deviant-standard) P3 (350–500 ms) in males and females in the acute stress state and neutral state.

The state main effect [*F*(1, 39) = 12.84, *p* < 0.05, *ηp^2^* = 0.25] was statistically significant. The amplitude of the P3 wave in both males and females was smaller in the stress state than in the neutral state. The state and the gender interaction [*F*(1, 39) = 0.27, *p* > 0.05, *ηp^2^* = <0.01] was not statistically significant. There was no statistically significant gender main effect [*F*(1, 39) = 0.04, *p* > 0.05, *ηp^2^* = <0.01]. See Table 5 and Figures 4, 5.

**Table 5:** Comparison of the difference wave (deviant-standard) P3 amplitude between males and females under acute stress and neutral state [(*x* ± *s*) μν].

	Females	Males	*F*	*P*	*ηp^2^*
Neutral	5.73 ± 1.02	5.26 ± 0.94	0.11	0.970	<0.01
Acute stress	4.08 ± 0.92	4.03 ± 0.85	<0.01	0.739	<0.01
*F*	7.85	5.06			
*P*	0.008	0.030			
*ηp^2^*	0.17	0.12			

## 4. Discussion

To verify the difference in the direction of effect of acute stress on behavioral inhibition in males and females, this study combined ERP techniques to understand the effect of evoked acute stress on behavioral inhibitory control in college students of different genders at the cognitive neural level. The results of this study found that the TSST paradigm was successful in eliciting stress state in subjects. In terms of stress results, the area under the stress state curve was larger in females than in males. From the behavioral data, the reaction time of females in both neutral and stress state was longer than that of males, and the number of errors of females in both neutral and stress state were more than that of males. These results suggest that there are differences in behavioral inhibitory control between males and females, and that females have relatively lower behavioral inhibitory control. Further analysis of ERP results showed that the N2 and P3 of both genders decreased as stress level increased, indicating that the increase in stress level could enhance the behavioral inhibitory control ability of individuals.

Regarding the N2 wave amplitude, it was found that the N2 wave amplitude decreased significantly in both males and females during the process from the neutral state to the stress state, while the variation in the wave amplitude was greater in females. The smaller amplitude of N2 suggests that acute stress promotes individual behavioral inhibitory control, which is consistent with previous studies (Silton et al., 2010; Clayson and Larson, 2013). For example, Rebecca reported that central frontal N2 wave amplitude was statistically significantly smaller in the emotional condition than in the neutral condition. It has been shown that larger N2 wave amplitude implies lower behavioral inhibition. For example, studies on PTSD patients have demonstrated that their low inhibition is associated with exhibiting larger N2 wave amplitude (Shu et al., 2014; Min et al., 2020), and studies on obese patients have confirmed the negative correlation between N2 wave amplitude and behavioral inhibition (Iceta et al., 2019). It is thus clear that evoked acute stress promotes behavioral inhibitory control in individuals.

Furthermore, the results showed greater changes in N2 wave amplitude in females during the process from neutral to stress state. This suggests that females have weaker conflict monitoring and conflict control under acute stress state, whereas males have an advantage in this regard, which is in line with previous studies. For example, a Go/No-go study of EEG recordings found females have a lower rate of correct responses and electrophysiological analyses suggest that females require more time for conflict detection as well as more resources for response execution (Melynyte et al., 2017). One reason for this is that female is more susceptible to external influences, more sensitive to stress and less able to regulate stress than male. Neuroimaging studies have shown that a decrease in the hippocampal response is associated with adaptive stress responses, while an increase in the hippocampal response is associated with non-adaptive stress responses (Sinha et al., 2016). In a study on gender differences in neurological stress responses, it was found that females had significantly higher bilateral hippocampal responses with increased dynamics than male under stress state, suggesting that females have more nonadaptive stress responses and less stress regulation than males under stress condition. Another reason for this difference may also be emotional influences. Kelly et al. administered the Visual Analogue Rating Scales and the Profile of Mood States after TSST stress. The results showed that the females were more timid, irritable, and confused and that females showed more pronounced subjective negative experiences under the same stress state (Kelly et al., 2008). At the same time, the hippocampal response was found to be higher in females in negative emotion studies (Stevens and Hamann, 2012), reflecting female’s deficiencies in negative emotion processing, such as stress dissipation.

Regarding P3 wave amplitude, it was found that P3 wave amplitude decreased significantly in both males and females during the process from neutral to stress state, and there was no significant difference in the variation of wave amplitude between females and males. A smaller amplitude of P3 suggests that acute stress promotes inhibitory control in individuals, which is consistent with previous research (Dierolf et al., 2017). It has been shown that the lower the P3 amplitude, the stronger the inhibitory control of the individual (Liu et al., 2020). Inhibitory control can be effectively improved and P3 amplitude reduced after training through inhibitory control (Melara et al., 2018). Typically, the P3 component reflects the process of assessing goals to achieve appropriate goal-directed responses (Kan et al., 2021). In the present study, there was no significant difference between females and males in the magnitude of variation in P3 wave amplitude during the process from the neutral to the stress state. This suggests that males and females under acute stress state invested approximately the same level of cognitive effort in the inhibition process itself. The reason for this may be that there is no significant gender difference between males and females at the time of late inhibition assessment and final decision making. This is in line with previous research. In a simple decision-making task, Weller et al. found no gender differences in making risky choices related to potential payoffs, with gender factors not playing a significant moderating role (Weller et al., 2010). Another reason for this result may also be due to the influence of educational background. This study selected college students as the subject group, and higher education factors may have contributed to the non-significant gender differences in decision making, which is consistent with previous studies. A behavioral study found no differences between males and females in the areas of risky decision making and inhibition in the experimental context (Kertzman et al., 2018).

In previous studies, the effects of acute stress on individual behavioral inhibition have diverged, speculating that the reason may be due to differences in the experimental and stress paradigms. In this study, compared to the Go/No-go paradigm and SST paradigm used in previous studies on behavioral inhibitory control, a two-choice Oddball paradigm is adopted to evoke behavioral inhibitory control function. It helps to analyze the two behavioral indicators of reaction time and correctness, and it can effectively avoid the interference of motor contamination on the results in ERP analysis, thus improving the interpretation of behavioral results and ERP results (Yuan Jiajin et al., 2017). In the selection of stressors, the TSST paradigm, which triggers psychological tension, is chosen to reduce the direct threat to the subject’s somatic body compared to the electric shock paradigm, which acts directly on the somatic body. According to a related description in dual-competition theory, when individuals are in a high-threat environment, high threat has a processing priority that consumes limited cognitive resources first, which in turn compromises processing resources for behavioral inhibition (Lim et al., 2008). When the level of environment threat is not high, a low threat environment enhances subjects’ arousal, enhances sensory sensitivity, helps to inhibit dominant responses, and promotes subjects’ behavioral inhibition (Pessoa, 2009).

The present findings also suggest some limitations and several directions for future research. First of all, only subjective emotion rating method was used to assess the stress state. Although the subjective assessment method is also a way to assess the stress state and is easy to operate, there are still individual subjective biases. Therefore, objective indicators, such as heart rate and cortisol, should be added in future studies. Secondly, only college students were selected as the subject group, and people of different ages could be invited to conduct the test in the future to improve the validity of the study results. Third, future plans could select fMRI techniques with higher resolution of neural activation data to further examine the effects of stress and gender on inhibitory function to validate and further develop the findings of this study. Finally, the present study did not fully consider the effects of other factors, such as personality traits and socioeconomic status, on the experimental results. Therefore, precise measurement and control of these variables are needed in future studies to avoid ambiguity in the interpretation of experimental results on the one hand, and to extend relevant research findings on the other.

## 5. Conclusion

In summary, evoked acute stress promoted behavioral inhibitory control in both males and females, and females were more sensitive to the stressful situation. In particular, acute stress reduced response inhibition time and response error rate, and decreased N2 and P3 wave amplitudes in college students of both genders. The change in N2 amplitude was greater in females when switching from neutral to stress state. Therefore, it is suggested that when individuals have sufficient cognitive resources, they should moderately increase tension to increase the level of physiological arousal and help improve behavioral inhibition, especially for the female group. A detailed examination of the acute stress and gender effects in behavioral inhibition processing and their interaction effects is beneficial to better understand the neural mechanisms of inhibition function. In the future, based on a deeper understanding of gender differences in inhibitory function, the development of gender-specific educational and neuropsychological intervention procedures can be explored to enhance behavioral inhibition more efficiently in both genders.

## Data availability statement

The raw data supporting the conclusions of this article will be made available by the authors, without undue reservation.

## Ethics statement

The studies involving human participants were reviewed and approved by the First Affiliated Hospital of Shihezi University, Shihezi University. The patients/participants provided their written informed consent to participate in this study.

## Author contributions

SD designed the experiment, collected data, and prepared the manuscript. XW collected the data and made data analysis. CM corrected the whole language of the manuscript and made final approval. LL gave technique supports and valuable suggestions in experiment designing. All authors contributed to the article and approved the submitted version.

## Funding

This research was supported by the Graduate Education Innovation Program of the Xinjiang Uygur Autonomous Region, China (XJ2022G097).

## Conflict of interest

The authors declare that the research was conducted in the absence of any commercial or financial relationships that could be construed as a potential conflict of interest.

## Publisher’s note

All claims expressed in this article are solely those of the authors and do not necessarily represent those of their affiliated organizations, or those of the publisher, the editors and the reviewers. Any product that may be evaluated in this article, or claim that may be made by its manufacturer, is not guaranteed or endorsed by the publisher.
